# Supplementary material for: Rapid detection of brucellosis using a quantum dot-based immunochromatographic test strip
Source: PLoS Negl Trop Dis. 2020 Sep 25;14(9):e0008557. doi: 10.1371/journal.pntd.0008557 (PMC7540878; doi:10.1371/journal.pntd.0008557)
Supplement: Attachment — Submitted filename: review.pdf [file pntd.0008557.s001.pdf]

The authors use fluorescent quantum dots as label in the development of an immunochromatographic lateral flow assay for human brucellosis

The manuscript should be improved at a number of minor points (see below). A main concern for this reviewer is that the authors claim a high sensitivity and specificity while the test is not validated against the accepted reference test which is culture. Instead the test was compared with RBT, SAT and an colloidal gold based LFA of which no reference is provided. Some attempts should be undertaken to test samples from culture confirmed patients

Other Comments

1. Introduction.
   a. The introduction is too generally and not focused on the relevant diagnostic issues such as the importance of validating newly developed Brucella tests against blood culture and refers to mostly old literature and ignoring recent good reviews of the different topics.
   b. a list of components used to make quantum dots is given which appears irrelevant here and it is not mentioned based on which criteria the components used in the present study was selected.
   c. the authors claim that quantum dots have important clinical applications without given any example in the context of diagnostics
   d. what is meant with that the developed method may be used as initial screening in epidemic areas. Why initial screening and why in epidemic areas (only?)?
   e. The authors state "that the detection limit of colloidal gold test strips for detecting proteins or small molecules is low" as an advantage of QD. However the results of the present study sees no difference between QD strips and colloidal gold strips. Why then this statement?
2. Method
   a. It may be mentioned that Brucella 104M is a B. abortus and for the non-expertise reader this might need some commenting as the authors mention B. melitensis as the more important human pathogen causing epidemics.
   b. Under "antigen preparation". How was the antigen concentration standardized, by which method and was any attempt done to qualitatively characterize the antigen? Did it contain any LPS?
   c. What is meant (line 129) with pipetted thoroughly and what is mentioned with "After coupling, blocking, resuspending and mixing" (line 130) the solution was diluted?
   d. Under 2.3.3 it is stated that After 10 min, the pad was observed using a portable lamp and a dry immunofluorescence analyser. Elsewhere, it is mentioned that the analysss was done by a handhold UV lamp. Is that different?
   e. Line 171. Three dilutions were tested (diluants?)?
   f. What type of machine was used to apply the test and control lines? Was this done by direct contact or by spraying. What function has the wiper? For the C line the solution was applied at 1.0 ug/cm. is this ug or ul?
   g. 2.3.4 Optimisation of test strip conditions is a micture of test strip optimization, test reaction optimization and stability testing and is confusing.
   h. 2.3.5 Method evaluating for screening. What was the cut-off value used for the SAT. Which GICA test was used and was this test validated against culture?

i. The origin and relevant characteristics of the patient samples should be described in detail.

3. Results
   A. how was the antigen concentration optimized to 3.9mg/ml?
   B. line 228 refers to fig 5 which is not included in the manuscript.
   C. Line 247 dimensions of binding pad should be given under the appropriate section (not here).
   D. 3.6 Optimisation of test strip construction. This section does not contain any optimasation contrary to what its title suggests.
   E. Line 308. States: “a T-line was not detected at a dilution of 256-fold,” however this reviewer sees a clear T-line at this dilution.
