# Supplementary material for: Rapid detection of brucellosis using a quantum dot-based immunochromatographic test strip
Source: PLoS Negl Trop Dis. 2020 Sep 25;14(9):e0008557. doi: 10.1371/journal.pntd.0008557 (PMC7540878; doi:10.1371/journal.pntd.0008557)
Supplement: Attachment — Submitted filename: response letter.docx [file pntd.0008557.s002.docx]

List of Responses

Dear Editors and Reviewers:

Thank you for your letter and for the reviewers’ comments concerning our manuscript entitled “**Rapid detection of brucellosis using a quantum dot-based immunochromatographic test strip**” (ID: **PNTD-D-20-00427**). The comments were all valuable and very helpful for revising and improving our paper, and provided important guidance for improving the significance of our research. We have studied the comments carefully and have made corrections that we hope will meet with your approval. The revised text is in red in the re-submitted paper. The main corrections in the paper and the responses to the reviewer’s comments are as follows:

**Responses to the reviewer’s comments:**

**1. Reviewer**

**Introduction:**

**a. Response to comment:** The introduction is too generally and not focused on the relevant diagnostic issues such as the importance of validating newly developed Brucella tests against blood culture and refers to mostly old literature and ignoring recent good reviews of the different topics.

**Response**: In this revised version of our manuscript, we have tried to improve the introduction section by adding information about the Chinese diagnostic criteria, which are based on serological and pathogenic tests. Serological testing with SAT is one of the standards that has been in use in China. Due to issues with biosafety and laboratory capacity, the cultivation method is only used in a small number of the top three hospitals and infectious disease hospitals.

**b. Response to comment**: a list of components used to make quantum dots is given which appears irrelevant here and it is not mentioned based on which criteria the components used in the present study was selected.

**Response:** In lines 69–77, we have briefly introduced the structure and composition of the quantum dots in order to familiarize readers with quantum dots. The type of QD selected in this study is the core-shell structure CdSe quantum dots. Compared with other structures, quantum dots are more stable and have better luminous performance. We have made changes in the text based on your comments.

**c. Response to comment**: the authors claim that QD have important clinical applications without given any example in the context of diagnostics.

**Response:** For evaluation of the QD test strip, a large portion of the serum samples collected were from clinical patients. Its efficiency was compared with that of SAT, RBT, etc., which are currently in use. It has high sensitivity in clinical screening for brucellosis, and the operation is very simple. Currently, only 199 serum samples were tested, but we will continue to evaluate the test strips later. In the revised manuscript, we have added information about quantum dot detection for MERS and swine fever to demonstrate the clinical application of QD.

**d. Response to comment**: what is meant with that the developed method may be used as initial screening in epidemic areas. Why initial screening and why in epidemic areas (only?)?

**Response:** As a test strip for the detection of brucellosis, our device has high sensitivity and good specificity, but the values are not 100% and false negatives and false positives may occur. Therefore, the results need to be confirmed with the help of a laboratory test such as SAT. The incidence of brucellosis in China is relatively high, and there are many endemic areas. Therefore, in order to prevent its spread, it needs to be monitored by screening tests, i.e., GICA and QD. In non-endemic areas, on the other hand, the number of cases is very small and patients can approach medical centers for diagnosis and treatment.

**e. Response to comment:** The authors state “that the detection limit of colloidal gold test strips for detecting proteins or small molecules is low” as an advantage of QD. However the results of the present study sees no difference between QD strips and colloidal gold strips. Why then this statement?

**Response**: In the case of the 199 serum samples tested in this study, the sensitivity and specificity of QD were higher than those of GICA. After the serum is diluted 256 times, QD detection can be performed 128 times and GICA detection can be performed 64 times. Further, the detection methods were analyzed in series and parallel and were found to be more efficient when they were used in series (the Youden index of sensitivity and specificity were 0.94 and 0.92, respectively).

**2.Reviewer Method:**

**a. Response to comment:** It may be mentioned that Brucella 104M is a B. abortus and for the non-expertise reader this might need some commenting as the authors mention B. melitensis as the more important human pathogen causing epidemics.

**Response:** In China, *B. melitensis* has become the predominant causative strain for brucellosis and shows strong virulence, but *B. aboutus* 104M is the earliest vaccine strain used in China. In addition, the virulence of 104M is relatively weak, and it shares the same antigenic determinant as *B. melitensis*, so the risk of using the vaccine is low.

**b. Response to comment:** Under “antigen preparation”. How was the antigen concentration standardized, by which method and was any attempt done to qualitatively characterize the antigen? Did it contain any LPS?

**Response:** We used Brucella 104M total protein, which was extracted and purified by a standard method, and then, we used a protein concentration analyzer for qualitative detection. The average value of three measured concentrations was used. At present, the antigen we used for labeling is whole bacterial protein. In our next work, we will label LPS and *Brucella* proteins of different species separately.

**c. Response to comment:** What is meant (line 129) with pipetted thoroughly and what is mentioned with “After coupling, blocking, resuspending and mixing” (line 130) the solution was diluted?

**Response:** We have explained these steps better in the revised manuscript. Briefly, all the reagents were mixed so that they could be completely activated. Coupling (binding of the quantum dot marker to antigens), blocking (addition of a blocking agent to the reaction), resuspension (addition of the original volume of resuspension solution), and mixing are the steps involved in quantum dot test strip labeling.

**d. Response to comment**: Under 2.3.3 it is stated that After 10 min, the pad was observed using a portable lamp and a dry immunofluorescence analyser. Elsewhere, it is mentioned that the analysss was done by a handhold UV lamp. Is that different?

**Response:** The portable lamp and handheld UV lamp refer to the same device. We apologize for the confusion. We have tried to clarify this point at various places in the manuscript. In this study, we used both qualitative and quantitative analysis. The T-line can be observed with a handheld UV lamp, and the light presented by the T-line can be digitized with a dry fluorescent immunoassay analyzer.

**e. Response to comment**: Line 171. Three dilutions were tested (diluants?)?

**Response:** Line 171 describes a simple treatment for the binding pad, and the purpose of this device is to improve the speed of chromatography and ensure that it is completed. We referenced these two processing methods for the binding pad from the previous literature.

**f. Response to comment**: What type of machine was used to apply the test and control lines? Was this done by direct contact or by spraying. What function has the wiper? For the C line the solution was applied at 1.0 ug/cm. is this ug or ul?

**Response**: The machine we used for spraying the C and T lines is a XY1000 dot film meter produced by Bio-Dot in the United States. The prepared solution is added to the spraying system according to the corresponding volume and sprayed by point contact. SPA and *Brucella* total bacterial protein is sprayed on the C and T lines, respectively; the C line spray volume is set to 1.0 μl/cm, and the T line spray volume is set to 0.6 μl/cm. We erroneously represented “μl” as “μg”. We apologize for this error, which has been corrected in the revised manuscript.

**g. Response to comment**: 2.3.4 Optimisation of test strip conditions is a micture of test strip optimization, test reaction optimization and stability testing and is confusing.

**Response**: We apologize. We have revised the description to elaborate on the preparation of test strips with regard to marking, coating, sealing, storing, and ensuring the stability of test strips. Specifically, we have described the determination of the ratio of QD, EDC, and NHS; the determination of the amount of antigen markers; the determination of the coupling time of QD and the whole protein, the amount and type of blocking agent, and the treatment of the binding pad; and the determination of the coating concentration. The storage temperature and reaction temperature of the paper strip were also analyzed.

**h. Response to comment**: 2.3.5 Method evaluating for screening. What was the cut-off value used for the SAT. Which GICA test was used and was this test validated against culture?

**Response**: The cut-off value used for SAT was a titer ≥ 1:100. This is a laboratory confirmed method that, together with clinical symptoms, is the gold standard for the diagnosis of brucellosis. In this study, we used SAT as the gold standard. Test strips are generally used for on-site testing and as a tool for initial screening. We collected the commercial GICA test strips, tested the same batch of serum samples with QD and GICA, and found that the sensitivity and specificity of QD were better than those of GICA.

**i. Response to comment**: The origin and relevant characteristics of the patient samples should be described in detail.

**Response**: These details have been added in the revised manuscript: this study involves a retrospective analysis of patients’ serum samples that were collected at the Tongliao Center for Endemic Disease between August 2018 and November 2019. Patients with a clear clinical history and experimental diagnosis were included. The diagnostic criteria were in strict accordance with the “Diagnostic Criteria for Brucellosis (WS 269-2019).”

**3.Reviewer**

**Results:**

**a. Response to comment**: how was the antigen concentration optimized to 3.9mg/ml?

**Response:** We selected Brucella 104M with better antigenicity for culture. The whole protein was used as the antigen, and the concentration after measurement was 3.9 mg/ml. This was used for coating the quantum dot test paper, and labelling the antigen and its concentration.

**b. Response to comment**: line 228 refers to fig 5 which is not included in the manuscript.

**Response:** We apologize for this error, which has been corrected. The content was moved to Figure 2 in our previous revision.

**c. Response to comment**: Line 247 dimensions of binding pad should be given under the appropriate section (not here).

**Response:** We have added detailed information in line 272.

**d. Response to comment**: 3.6 Optimisation of test strip construction. This section does not contain any optimasation contrary to what its title suggests.

**Response:** We wanted to explain the assembly process for the test strip. Therefore, we have revised the title to “3.6. Assembly of the test strip.”

**Other changes:**

1. Line 157, Figure 1 depicts the composition of the test strip. Lines 148–156 explain the principle of chromatography in detail.

2. “NTC film/membrane” has been revised to “NC membrane” in lines 185, 257, 278, and 384.

We have tried our best to improve the manuscript based on the comments provided, but these changes do not influence the content and framework of the paper. Here, we have not listed the changes but we have indicated them with red text in the revised paper. We appreciate the editor’s and reviewers’ work on our manuscript, and we hope that the revised manuscript will meet with everyone’s approval.

Once again, thank you very much for your comments and suggestions.
